# A high-throughput 3’ UTR reporter screening identifies microRNA interactomes of cancer genes

**DOI:** 10.1371/journal.pone.0194017

**Published:** 2018-03-09

**Authors:** Gert Van Peer, Evelien Mets, Shana Claeys, Ines De Punt, Steve Lefever, Maté Ongenaert, Pieter Rondou, Frank Speleman, Pieter Mestdagh, Jo Vandesompele

**Affiliations:** Center for Medical Genetics, Department of Pediatrics and Genetics, Ghent University, Ghent, Belgium; Universitat des Saarlandes, GERMANY

## Abstract

**Introduction:**

Despite the established contribution of deregulated microRNA (miRNA) function to carcinogenesis, relatively few miRNA-cancer gene interactions have been validated, making it difficult to appreciate the true complexity of miRNA-cancer gene regulatory networks.

**Results:**

In this effort, we identify miRNA interactomes of 17 well-established cancer genes, involved in various cancer types, through a miRNome-wide 3’ UTR reporter screening. Using a novel and performant strategy for high-throughput screening data analysis, we identify 390 interactions, quadrupling the size of the known miRNA interactome for the cancer genes under investigation. Clear enrichments of established and predicted interactions underscore the validity of the interactome data set. Interactomes appear to be primarily driven by canonical binding site interactions. Nonetheless, non-canonical binding sites, such as offset 6mer and seed-mismatched or G:U wobble sites, also have regulatory activity, albeit clearly less pronounced. Furthermore, we observe enhanced regulation in the presence of 3’ supplementary pairing for both canonical and non-canonical binding sites.

**Conclusions:**

Altogether, the cancer gene-miRNA interactome data set represents a unique resource that will aid in the unraveling of regulatory miRNA networks and the dynamic regulation of key protein-coding cancer genes. In addition, it uncovers aspects of the functional miRNA binding site’s architecture and the relative contributions of different binding site types.

## Introduction

In normal cells, the expression of tumor suppressor genes and oncogenes is tightly controlled by a myriad of cooperative genetic and epigenetic mechanisms to guarantee correct dynamic gene dosages. Perturbation of these mechanisms can result in aberrant expression and may contribute to cancer formation. Post-transcriptional regulation by microRNAs (miRNAs) is one of the best-characterized gene regulatory mechanisms, and deregulated miRNA expression has been extensively documented in the pathogenesis of various cancer types [1]. While evidence emerged that miRNAs can work in oncogenic or tumor suppressor cooperative networks [2–4], aberrant expression of even a single miRNA can be sufficient to initiate tumor development [5,6].

miRNAs are small non-coding RNA molecules with a length of approximately 21 nucleotides. Mature miRNAs are processed from precursor molecules and execute their gene regulatory function by guiding an effector complex, the miRNA-induced silencing complex (miRISC), to binding sites in target mRNA molecules [7]. Upon binding, miRISC initiates a sequence of events leading to inhibition of translation and decay of mRNA molecules, and ultimately to reduced protein levels [8–10].

Complementarity with the miRNA seed region, the sequence spanning nucleotides 2 to 7 of the 5’ end of the mature miRNA, appears to be the most important determinant of a functional miRNA binding site in vertebrates (Figure A in S1 Fig) [11–13]. Not surprisingly, the seed-region is the most evolutionarily conserved region of miRNAs [11,14]. Frequently, 6mer seed-pairing is augmented with an adenosine at the 3’ end of the site, constituting a 7mer-A1 binding site. Similar to the seed-match, the presence of adenosines at this position is highly evolutionary conserved [12,15]. Despite clear conservation, sites with a nucleotide match instead of an adenosine have also occasionally been described to be functional [16]. Alternatively, 6mer seed-pairing can be preceded by an additional nucleotide match at the 5’ end of the site, constituting a 7mer-m8 binding site. If both the 3’ adenosine and the additional 5’ match are present, an 8mer binding site is established. On average, 8mer sites are more efficacious than 7mer-m8 sites, which in turn are more efficacious than 7mer-A1 sites and 6mer sites respectively (Figure A in S1 Fig). Sequence complementarity to the 3’ end of the miRNA, or so-called 3’ supplementary binding (Figure B in S1 Fig), has been shown to slightly increase seed-matched site potency [13,15,17]. Seed-matched miRNA binding sites have typically been considered as canonical sites, being both more frequently involved in miRNA interactions and mediating more pronounced regulation compared to other site types. Both statements, however, are still being debated and contradictory reports exist.

In addition to seed-matched sites, non-canonical binding has been described, but only a limited number of efforts have delineated well-defined non-canonical binding site patterns. Offset 6mer sites represent one class of non-canonical sites and display a seed-match with a single-nucleotide offset (Figure C in S1 Fig) [12,13,16,18,19]. Seed-mismatched sites represent another type of non-canonical sites and have a single nucleotide mismatch in the seed region (Figure D in S1 Fig) [20,21], or a G:U wobble [21,22], which is an energetically more favorable mismatch. The imperfect seed-match of these sites is sometimes compensated by extensive 3’ compensatory pairing (Figure B in S1 Fig), although such sites are rather rare [13,23]. Centered sites are characterized by at least 11 consecutive nucleotide matches to the central region of the miRNA (either nucleotides 4–14 or 5–15), without substantial pairing to the 5’ or the 3’ ends of the miRNA [11,24]. G-bulge sites are also seed-mismatched, but a nucleotide is bulged out in the mRNA in order to match the seed-region (Figure E in S1 Fig) [11,14,25]. Occasionally, miRNA-mRNA interactions with seed-mismatches, but with extensive pairing along the entire mRNA have been observed [12,15,26,27].

In general, non-canonical binding sites appear to be less potent than canonical sites, although there is ongoing debate. Offset 6mer, G-bulge and seed-mismatched or G:U wobble sites are thought to be either not effective or less effective than 6mer sites [19,20,25]. Centered sites and 3’ compensatory sites, on the other hand, have clearly been shown to have a regulatory effect, but are thought to constitute less than 1% of all targeting [23,24]. Varying numbers have been reported on the prevalence of non-canonical interactions, ranging from as low as 7% to as high as 88% [20,21,25,28,29]. Hence, further large-scale studies are warranted to investigate both the prevalence and the potency of non-canonical binding events.

Initially, miRNA binding sites were thought to be exclusively located in the 3’ untranslated region (UTR) of mRNA molecules. However, functional miRNA binding sites have occasionally been reported in 5’UTRs [16,30] and, more frequently, within mRNA coding sequences [17,31,32]. Recently, large-scale mappings of miRNA interactions with AGO CLIP-seq based methods and AGO CLASH have supported this notion and revealed miRNA binding to the entire length of mRNA molecules [13,15,17,28,29]. Notably, Helwak et al. observed the largest number of miRNA interactions with mRNAs to occur in the coding sequence (61%), followed by the 3’ UTR (34%) and 5’UTR (5%) [28]. In contrast, Chi et al. observed the majority of miRISC-binding to occur in 3’ UTRs of mRNAs (61%), followed by the coding sequence (38%) and the 5’UTR (1%) [33]. A recent meta-analysis of 34 AGO CLIP-seq data sets by Clark et al. confirmed the latter finding [29]. Of note, the relative proportion of binding events in 5’UTRs, coding sequences, and 3’ UTRs varies between individual miRNAs [28]. Despite ongoing debate, the regulatory effect of miRNAs is believed to be mainly attributable to 3’ UTR interactions. Interactions outside the 3’ UTR seem to confer little regulatory activity [20,21,34,35] and potentially mediate more subtle regulation or serve other functions.

Typically, miRNAs have an extensive target repertoire, with estimated averages of 100 to 1000 target sites per miRNA, and with multiple sites often present per mRNA [13,23,29]. In addition, mRNAs are frequently targeted by more than one miRNA [36]. Up to half of the human protein-coding genes are believed to be controlled by miRNAs [19]. Nonetheless, only few miRNA interactions are actually validated, making it difficult to appreciate the true complexity of miRNA regulation. Furthermore, reported interactions are often validations of model predictions, and are therefore biased towards interactions adhering to the current (incomplete) rules describing miRNA binding. Hence, important regulatory miRNAs with a non-canonical mode of interaction are potentially ignored. Knowledge on the full complement of regulatory miRNAs is imperative to understand the dynamic regulation and potential deregulation of genes in disease and development.

Here, we identify the miRNA interactomes of a set of 17 established cancer genes, involved in various cancer types. Applying an unbiased, miRNome-wide 3’ UTR reporter screening, we identify 390 interactions, quadrupling the available knowledge on miRNA regulation for these genes. We show that miRNA interactomes appear to be primarily driven by canonical binding site interactions. However, non-canonical binding sites also confer regulation, albeit clearly less pronounced. Furthermore, enhanced regulatory activity upon 3’ supplementary binding is present for both canonical and non-canonical binding sites.

## Results

**Fig 1:**
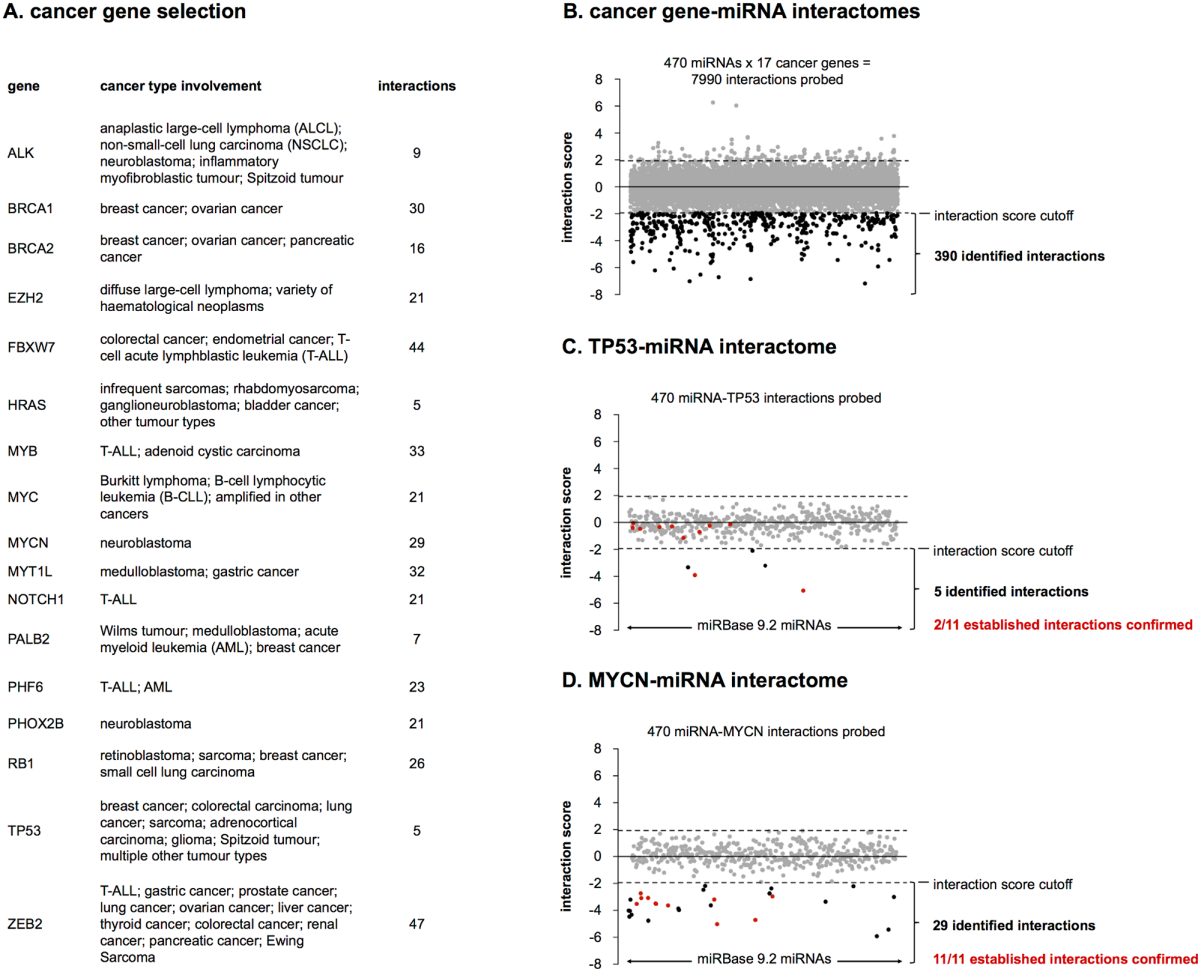
Cancer gene-miRNA interactomes. **(A)** Selection of 17 cancer genes involved in multiple cancer types. For each gene the number of interactions identified in the 3’ UTR reporter screening is listed. **(B)** Overview of 3’ UTR reporter screening results. Average interaction scores for all probed miRNA-3’ UTR combinations. **(C)** The miRNA interactome of TP53. **(D)** The miRNA interactome of MYCN.

miRNA interactomes were inferred for 17 genes with known pan-cancer involvement or an established role in cancer types such as breast cancer, lung cancer, colon cancer, T-cell acute lymphoblastic leukemia (T-ALL) and neuroblastoma (Fig 1A). The cancer gene selection is supported by information from the Cancer Gene Census (cancer.sanger.ac.uk/cancergenome/projects/census/) and selected publications (see S1 Table for PubMed IDs).

Interactions between all miRNAs annotated in miRBase 9.2 and the 3’ UTRs of the selected genes were probed in independently replicated reporter gene screenings. In brief, HEK293T cells were co-transfected with 3’ UTR luciferase reporter constructs and a library of 470 miRNA mimics, in total probing 7990 interactions. Forty-eight hours after co-transfection, reporter gene activities were assessed to score potential down-regulation as a result of miRNA-3’ UTR interaction. Reporter gene activities were converted into interaction scores to eliminate miRNA-treatment-specific bias. An interaction score threshold was then applied in order to label interactions.

### A novel metric for the scoring of high-throughput screening data

Interactions between miRNAs and 3’ UTRs were evaluated calculating the interaction score, a novel metric for the analysis of high-throughput screening data. Analysis of reporter gene activities with classic z-score calculation revealed a miRNA-specific bias in screening results, with unequal distributions of z-scores for different miRNAs (S2 Fig). Under the assumption that none of the tested miRNAs systematically targets a substantial part of all genes investigated, the interaction score eliminates the systematic bias through median centering of miRNA-specific z-score distributions. The interaction score is more negative for miRNAs that interact with the 3’ UTR.

**Fig 2:**
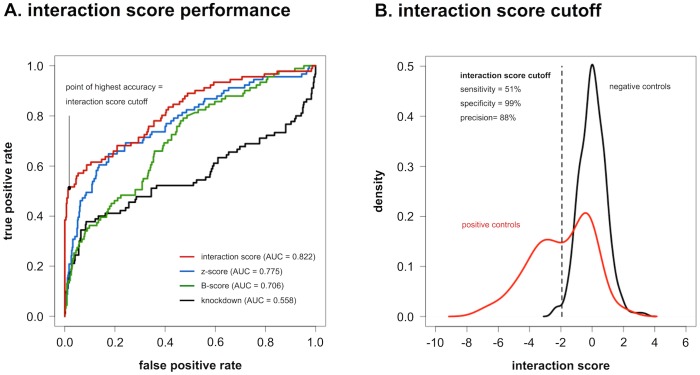
Interaction score performance. **(A)** ROC-curve analysis of different metrics for high-throughput screening data-analysis on a set of positive and negative controls in the 3’ UTR reporter screening. Interaction scores and z-scores are calculated as described in the Methods section. B-scores are obtained by applying Tukey median polish to z-scores, in order to remove plate positional bias. Knockdowns are calculated by expressing normalized reporter activities (NRAs) relative to the average NRA of four non-targeting miRNA treated controls in the same assay plate. **(B)** Distributions of average interaction scores for positive and negative controls are clearly distinct. Application of the interaction score cutoff retrieves positive controls with 51% sensitivity, whereas negative controls are correctly called with a specificity of 99%. Precision obtained with this cutoff (i.e. the proportion of identified interactions that are true interactions) is 88%. Reprinted from Van Peer et al. [37] under a CC-BY 4.0 license, with permission from Oxford University Press, original copyright 2016.

In this data set, the interaction score outperforms commonly used metrics for high-throughput screening data analysis, such as z-scores, B-scores and knockdown percentages. This is apparent from ROC-curve analysis, using a set of previously reported interactions present in our screening as positive controls, and a set of interaction scores from an empty 3’ UTR reporter screening as negative controls (Fig 2A). Areas under the ROC-curve (AUC) are significantly different for the different metrics (p < 0.05), with the interaction score having the best overall performance (AUC = 0.822). The point of highest accuracy in this ROC-curve (interaction score = -1.94; accuracy = 91%) was put forward as the interaction score cutoff to separate positive from negative interactions, and corresponds to a precision of 88%, a specificity of 99% and a sensitivity of 51%, hereby favoring false negative over false positive interactions.

### High technical and biological reproducibility of 3’ UTR reporter screenings

**Fig 3:**
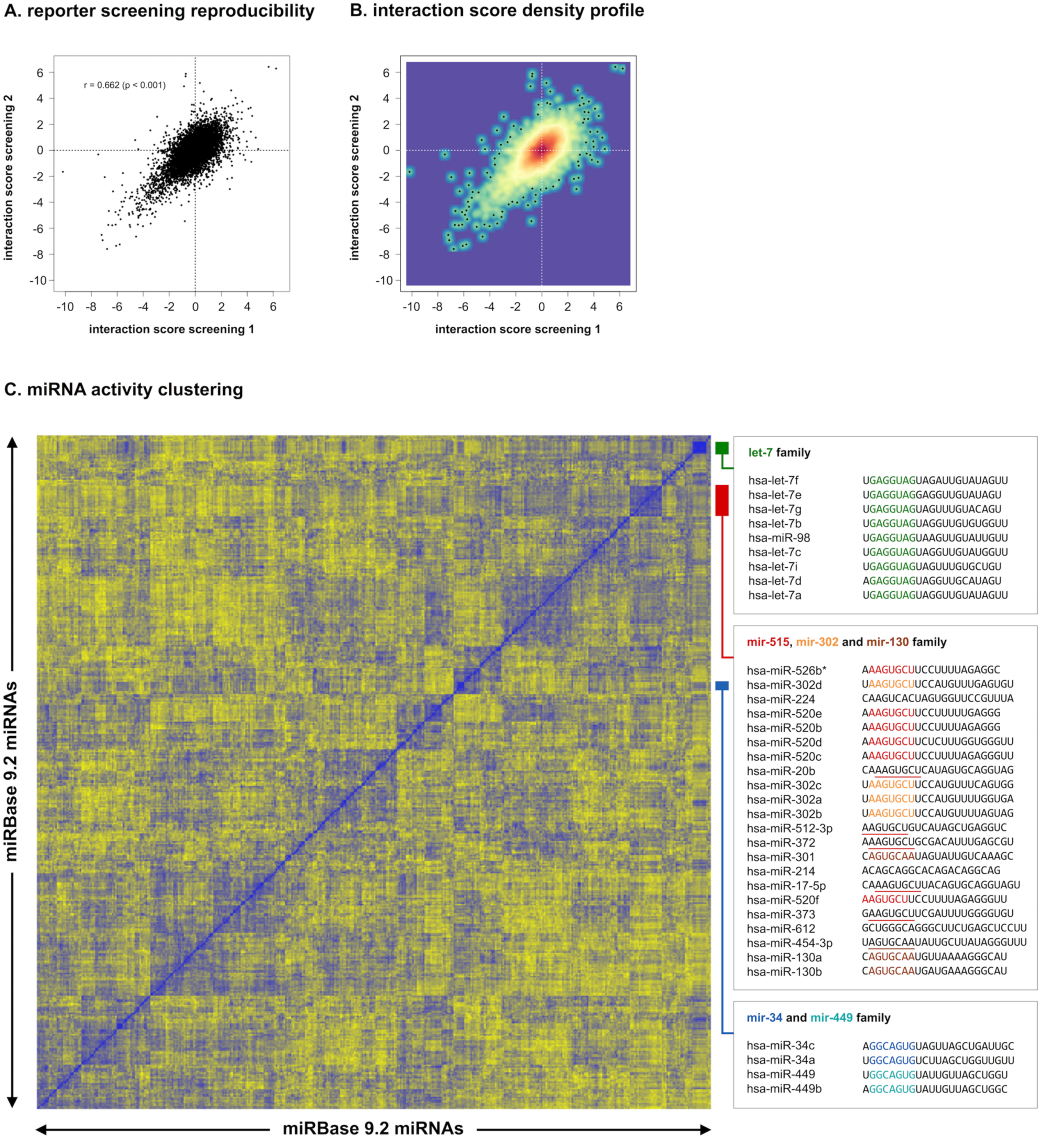
3’ UTR reporter screening reproducibility. **(A)** Correlation of interaction scores from replicate 3’ UTR reporter screenings. **(B)** Correlation of interaction scores with a density profile, showing that the largest fraction of interaction scores is centered around 0. **(C)** Hierarchical clustering of miRNAs according to their activity in the 3’ UTR reporter screening. For each miRNA pair, the Pearson correlation between average interaction scores for all 17 cancer genes was calculated. Correlation vectors for all miRNAs are subsequently clustered using Euclidean distance as the distance measure. Members of the same miRNA family, in addition to families with identical or similar seed-sequences, cluster together. For the let-7 family, 9 out of 9 members cluster together. The mir-34 (2 out of 3 members) and the mir-449 family (2 out of 2 members) also cluster together. The only member not clustering (hsa-miR-34b) is the only one having a different, 1-nucleotide offset seed sequence. The mir-302 (4 out of 5 members) and mir-515 family (6 out of 32 members) cluster together with miRNAs with identical or 1-nucleotide offset seed sequences (red underline) such as hsa-miR-20b, hsa-miR-512-3p, hsa-miR-372, hsa-miR-373 and hsa-miR-17-5p. The mir-130 family (3 out of 3 members) clusters together with hsa-miR-454-3p that has an identical seed sequence (brown underline).

For each gene, either duplicate (ALK, BRCA1, BRCA2, EZH2, FBXW7, HRAS, MYB, MYC, MYCN, MYT1L, NOTCH1, PALB2, PHOX2B, RB1 and ZEB2) or triplicate (PHF6 and TP53) 3’ UTR reporter screenings were performed. Reproducibility of replicated screenings was high, as can be appreciated from the correlation in interaction scores (Pearson correlation = 0.662, p < 0.05) (Fig 3A and 3B). Further underscoring this reproducibility is the observation that similar miRNA sequences display similar regulatory behavior, as apparent from the clustering of miRNA family members according to their activity in the screening (Fig 3C). Prominent examples are the let-7 family (miRNA family ID: MIPF0000002) and the mir-130 family (MIPF0000034), of which respectively all nine and all three mature miRNAs annotated in miRBase 9.2 cluster together. Furthermore, different miRNA families with identical seed sequence, such as the mir-34 (MIPF0000039) and the mir-449 family (MIPF0000039), or the mir-302 (MIPF0000071) and mir-515 family (MIPF0000020), also cluster together.

### Enrichment of predicted and established interactions

**Fig 4:**
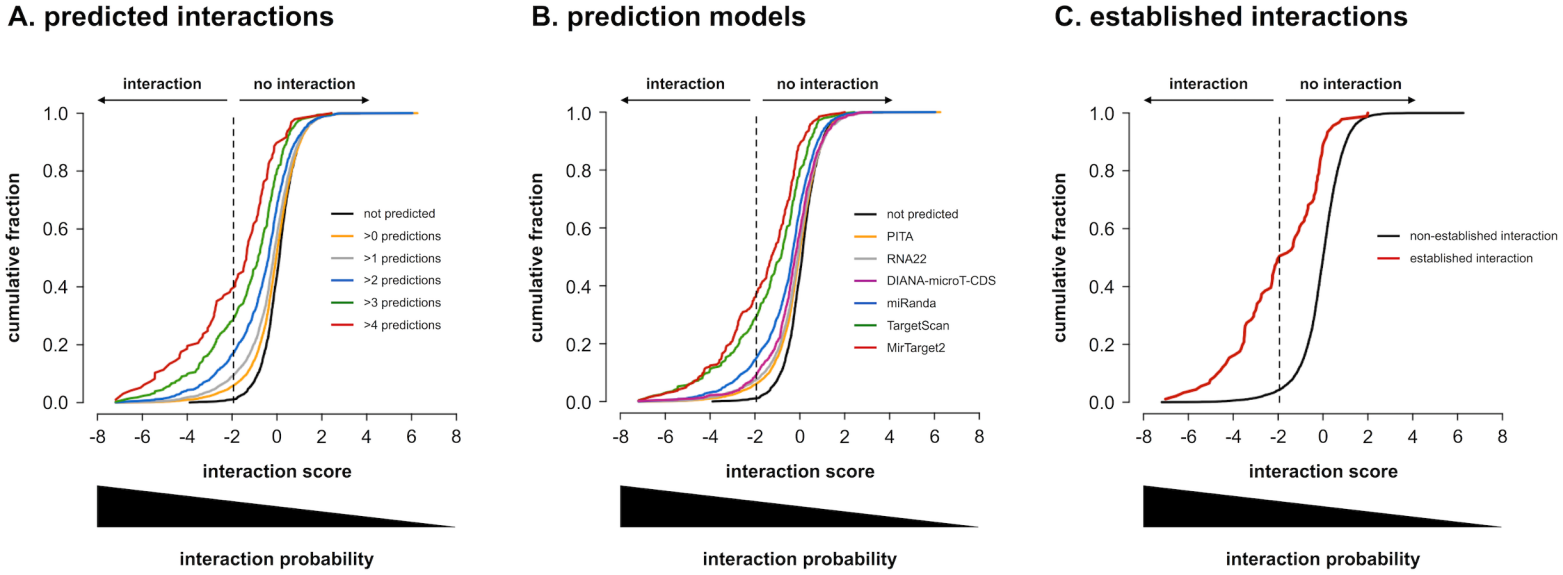
Predicted and established interactions. Cumulative distributions of average interaction scores for all 7990 miRNA-3’ UTR combinations probed. **(A)** according to the number of models that predict them as true interactions. Interaction scores are clearly lower for combinations that are predicted by more models. All distributions are significantly different from one another (one-sided Kolmogorov-Smirnov p-values < 0.01 after Benjamini-Hochberg multiple testing correction). **(B)** according to prediction by individual models. MirTarget2 predictions have the lowest scores. For each model, the distribution of interaction scores for predicted interactions is significantly different from that of non-predicted interactions (one-sided Kolmogorov-Smirnov p-values < 0.01 after Benjamini-Hochberg multiple testing correction). **(C)** according to whether they have previously been established as true interactions or not. Previously established interactions clearly have lower interaction scores. Distributions are significantly different (one-sided Kolmogorov-Smirnov p-value < 0.001).

Predicted miRNA-3’ UTR interactions have significantly more negative interaction scores in our data set. Moreover, score distributions gradually shift towards more negative values as more models predict the interactions (Fig 4A, p < 0.01). Combining the output of multiple models has been questioned in the past [38], but seems to be able to increase the precision of prediction (also referred to as the positive predictive value) in our data set (precision = 39% for predictions by at least five models). While all tested models (TargetScan, Mirtarget2, PITA, RNA22, miRanda and DIANA-microT-CDS) yielded interaction score distributions that were significantly shifted towards more negative scores, it is clear that different models have different performances, with MirTarget2 having the highest precision (37%) and the most pronounced shift (Fig 4B). Similar to predicted interactions, previously established interactions (see ‘Interaction score calculation’ in Methods section for details) also have significantly more negative interaction scores (Fig 4C, p < 0.001), further underscoring the validity of our data set.

### Identification of cancer gene miRNA interactomes

**Fig 5:**
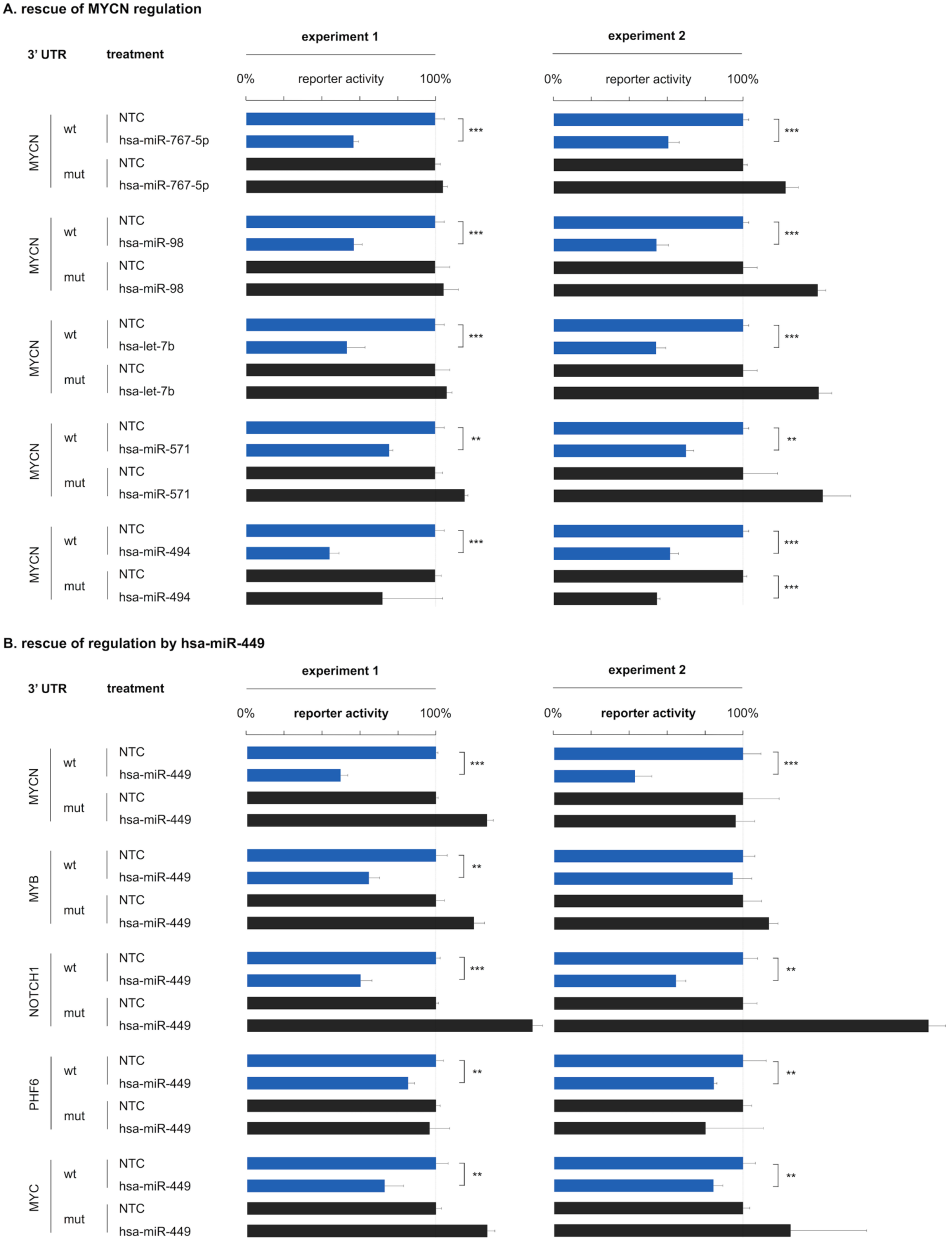
3’ UTR reporter rescue experiment. Rescue of 3’ UTR reporter regulation. **(A)** For four MYCN interactions significant down-regulation of reporter activity after miRNA expression modulation can no longer be demonstrated upon canonical binding site mutation (one-sided t-test; p < 0.001 ***; p < 0.01 **; wt = wild-type 3’ UTR; mut = mutant 3’ UTR) in two independently replicated reporter experiments. Reporter activity is expressed relative to non-targeting miRNA treated controls (NTC). Error bars represent standard deviations on three technical replicates. Successful rescue of MYCN regulation could only be achieved in one experiment for hsa-miR-494. **(B)** For four hsa-miR-449 interactions significant down-regulation of reporter activity after miRNA expression modulation can no longer be demonstrated upon canonical binding site mutation (one-sided t-test; p < 0.001 ***; p < 0.01 **; wt = wild-type 3’ UTR; mut = mutant 3’ UTR) in two independently replicated reporter experiments. Reporter activity is expressed relative to non-targeting miRNA treated controls (NTC). Error bars represent standard deviations on three technical replicates. Successful rescue of regulation by hsa-miR-449 could only be achieved in one experiment for MYB.

Applying the highly specific and precise interaction score cutoff, we identified miRNA interactomes of 17 selected cancer genes. A total of 390 interactions was identified, of which 344 are novel (Fig 1B). Notably, 83 of the identified interactions (21%) lack a seed-match and are therefore not detected by most target prediction models, emphasizing the power of an unbiased approach. A comprehensive overview of screening results and the miRNA interactomes of individual cancer genes are presented in S2 Table and S3 Fig. As representative examples, we focus on the interactomes of TP53 and MYCN, respectively the best-established tumor suppressor gene and one of the few genes included in our screening effort with a substantial number of previously established miRNA interactions. For TP53, we identified five interactions of which two were previously reported (Fig 1C). Nine previously reported interactions could not be confirmed in our screening, which may be due to the nature of the interaction score cutoff, favoring false negatives over false positives. Another possible explanation is that this may partly represent a positive publication bias for the most widely studied cancer gene. For MYCN, we could confirm all 11 previously reported interactions and in addition identified 18 novel interactions (Fig 1D). For four out of five randomly selected, novel interactions with MYCN, we were able to abrogate regulation upon mutation of canonical binding site patterns in two independently replicated experiments (Fig 5A). Similarly, for four out of five genes (MYCN, NOTCH1, PHF6, MYC) regulation by hsa-miR-449 could be abrogated in two independently replicated experiments (Fig 5B).

### Regulation of endogenous mRNA levels

**Fig 6:**
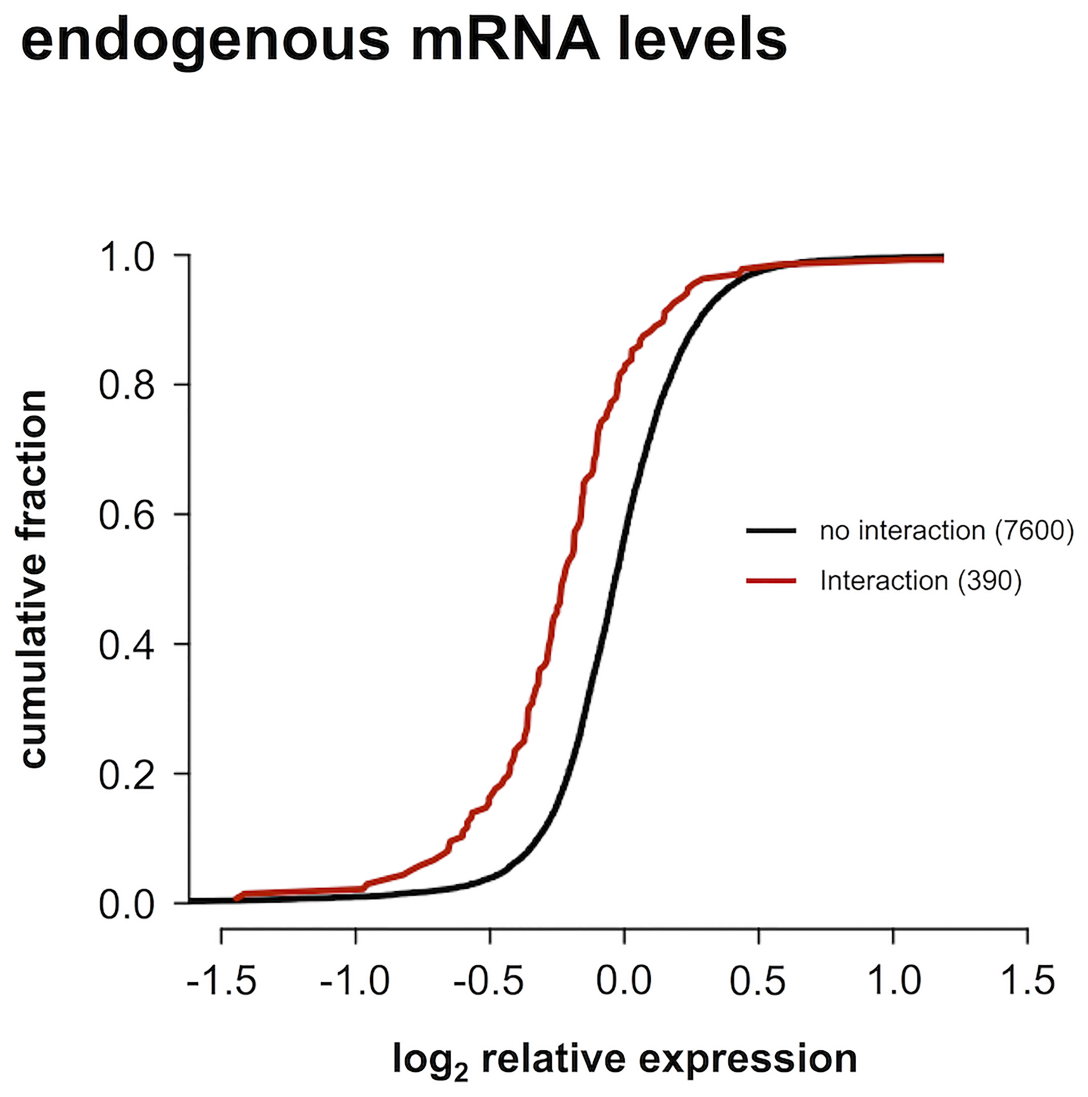
Endogenous mRNA levels. Cumulative distributions of log_2_ relative expression levels of endogenous mRNAs measured with RT-qPCR after miRNA modulation. The distribution for interactions identified in the 3’ UTR reporter screening is significantly lower than that for miRNA-3’ UTR combinations for which no interaction was observed (one-sided Kolmogorov-Smirnov p-value < 0.001).

In order to validate the interactome data set, we used RT-qPCR to measure endogenous mRNA levels for the 17 target genes fourty-eight hours after modulation with 470 miRNA mimics. While an RT-qPCR readout has the advantage of probing endogenous transcript levels, it will not detect any effects resulting from translational inhibition. Nonetheless, we observed significantly lower expression of endogenous mRNAs for the 390 interactions identified in the 3’ UTR reporter screening, than for the 7600 miRNA-3’ UTR combinations for which no interaction was found (Fig 6, p < 0.001).

### Canonical binding site potency

**Fig 7:**
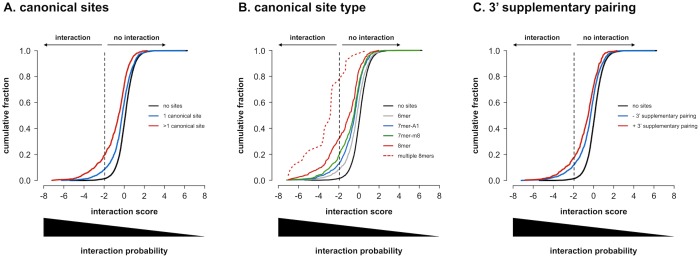
Canonical binding site potency. Cumulative distributions of average interaction scores for all 7990 miRNA-3’ UTR combinations probed. **(A)** according to the presence of canonical binding site patterns. Combinations with multiple canonical binding site patterns have lower interaction scores than combinations with a single pattern, that in their turn have lower scores than combinations without canonical binding site patterns. All distributions are significantly different from one another (one-sided Kolmogorov-Smirnov p-values < 0.001 after Benjamini-Hochberg multiple testing correction). **(B)** according to the presence of different types of canonical binding site patterns. Combinations with at least one 8mer pattern, have lower interaction scores than combinations with at least one 7mer-m8, one 7mer-A1 and one 6mer pattern, respectively (combinations with multiple types of binding site patterns are considered in all respective distributions). Notably, the presence of multiple 8mer patterns produces the largest shift in distribution. All distributions are significantly different from one another (one-sided Kolmogorov-Smirnov p-values < 0.01 after Benjamini-Hochberg multiple testing correction). **(C)** according to the presence of 3’ supplementary pairing. Combinations harboring canonical binding site patterns with 3’ supplementary pairing have lower interaction scores than those without. All distributions are significantly different from one another (one-sided Kolmogorov-Smirnov p-values < 0.001 after Benjamini-Hochberg multiple testing correction).

miRNA-3’ UTR combinations with canonical binding sites (2456 combinations with 3730 sites) have significantly more negative interaction scores than combinations without (Fig 7A, p < 0.001). In addition, combinations with multiple canonical sites (818 combinations) have more negative scores as compared to combinations with only a single canonical site (p < 0.001). Furthermore, the hierarchy in potency of the different canonical binding sites is reflected in the data, with 8mer sites (263 combinations with 298 sites) being the most potent, followed by 7mer-m8 (665 combinations with 746 sites), 7mer-A1 (699 combinations with 802 sites) and 6mer sites (1473 combinations with 1884 sites) (Fig 7B, p < 0.01 for each comparison). Remarkably, merely looking at the presence of multiple 8mer sites (26 combinations) predicts negative interaction scores with higher precision (77%) than any of the prediction models considered or combination thereof. In the presence of 3’ supplementary pairing, the distribution of scores for combinations with canonical binding sites (488 combinations with 531 sites) shifts towards more negative values, confirming that 3’ supplementary pairing increases canonical binding site potency (Fig 7C, p < 0.001).

### Non-canonical binding site potency

**Fig 8:**
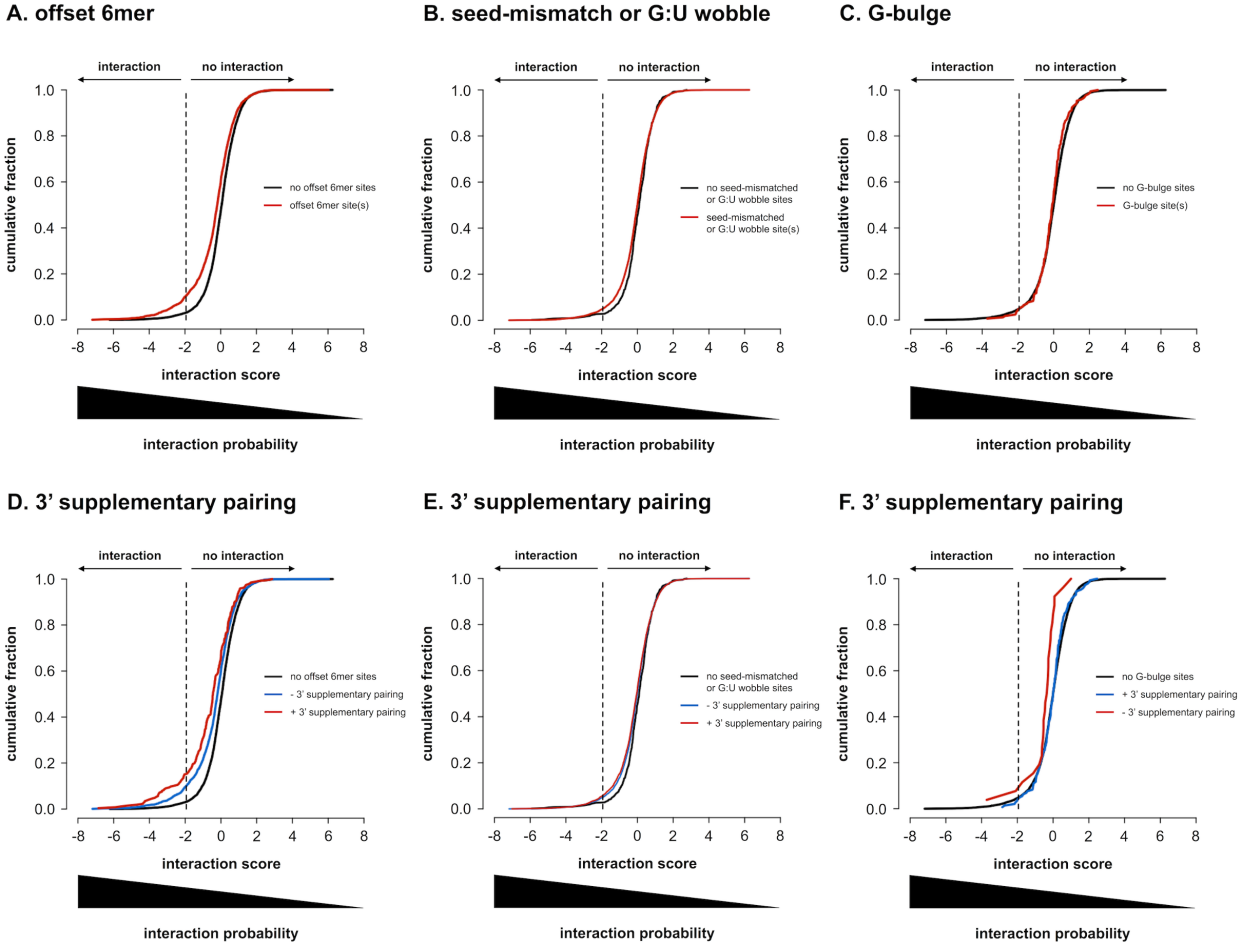
Non-canonical binding site potency. Cumulative distributions of average interaction scores for all 7990 miRNA-3’ UTR combinations probed. **(A)** according to the presence of offset 6mer binding site patterns. Combinations with at least one offset 6mer pattern, have lower interaction scores than combinations without. Distributions are significantly different (one-sided Kolmogorov-Smirnov p-value < 0.001). **(B)** according to the presence of seed-mismatched or G:U wobble binding site patterns. Combinations with at least one seed-mismatched or G:U wobble pattern have lower interaction scores than combinations without. Distributions are significantly different (one-sided Kolmogorov-Smirnov p-value < 0.01). **(C)** according to the presence of G-bulge binding site patterns. Combinations with G-bulge patterns don’t have detectably lower interaction scores than combinations without. Distributions are not significantly different (one-sided Kolmogorov-Smirnov p-value > 0.05). **(D)** according to the presence of offset 6mer binding site patterns with 3’ supplementary pairing. Combinations harboring offset 6mer patterns with 3’ supplementary pairing have lower interaction scores than those without. All distributions are significantly different from one another (one-sided Kolmogorov-Smirnov p-values < 0.001 after Benjamini-Hochberg multiple testing correction). **(E)** according to the presence of seed-mismatched or G:U wobble binding site patterns with 3’ supplementary pairing. Combinations harboring seed-mismatched or G:U wobble patterns with 3’ supplementary pairing have lower interaction scores than those without. All distributions are significantly different from one another (one-sided Kolmogorov-Smirnov p-values < 0.05 after Benjamini-Hochberg multiple testing correction). **(F)** according to the presence of G-bulge binding site patterns with 3’ supplementary pairing. Combinations harboring G-bulge patterns with 3’ supplementary pairing have lower interaction scores than those without. All distributions are significantly different from one another (one-sided Kolmogorov-Smirnov p-values < 0.05 after Benjamini-Hochberg multiple testing correction).

The contradictory evidence regarding the regulatory potential of non-canonical binding sites prompted us to evaluate them in our interactome data set. For offset 6mer sites (1863 combinations with 2514 sites), we observe a clear regulatory effect, with a significant shift in the distribution of interaction scores (Fig 8A, p < 0.001). Seed-mismatched or G:U wobble sites (7466 combinations with 55975 sites) also have regulatory potential, although the shift in distribution is clearly less pronounced (Fig 8B, p < 0.01). Furthermore, no significant differences in distribution could be observed for sites with the G:U wobble or mismatch at a particular position in the seed region, suggesting that no preferential position exists (data not shown; Kolmogorov-Smirnov p-values > 0.05 after Benjamini-Hochberg multiple testing correction). Also, no difference between a G:U wobble, which is an energetically more favorable mismatch, and other mismatches could be observed (data not shown; Kolmogorov-Smirnov p-values > 0.05 after Benjamini-Hochberg multiple testing correction). G-bulge sites (170 combinations with 177 sites) don’t appear to have any regulatory potential in this data set and the score distribution of miRNA-3’ UTR combinations with G-bulge sites is not different from combinations without G-bulge patterns (Fig 8C, p > 0.05). Centered sites were too low in abundance (6 combinations with 6 sites) to have enough statistical power to detect subtle regulatory activity.

Similar to canonical binding sites, non-canonical binding sites seem to be more potent in the presence of additional binding to the 3’ end of the miRNA. For offset 6mer sites, the additional effect is significant, with a pronounced shift in the distribution of interaction scores (266 combinations with 275 sites) (Fig 8D, p < 0.001). For seed-mismatched or G:U wobble sites, the added effect of 3’ supplementary binding is significant but small and therefore probably biologically less relevant (3973 combinations with 7608 sites) (Fig 8E, p < 0.05). For seed-mismatched or G:U wobble sites, 3’ compensatory binding has been described as a more extensive form of 3’ supplementary binding, compensating the incomplete seed-match. In this data set, however, the effect of 3’ compensation and 3’ supplementation could not be distinguished (data not shown; Kolmogorov-Smirnov p-value > 0.05). G-bulge sites at last, although having no apparent effect in the absence of 3’ supplementary pairing, seemed to have modest regulatory activity in its presence (26 combinations with 27 sites) (Fig 8F, p < 0.05).

## Discussion

We defined miRNA interactomes of 17 cancer genes involved in multiple cancer entities, based on an unbiased 3’ UTR reporter screening of unprecedented scale, probing 470 miRNAs. With 390 interactions identified (of which 344 novel) and 92 a priori known interactions, we quadrupled the size of the known miRNA interactome for these genes. To analyze the screening results, we developed a novel metric, the interaction score, that outperforms commonly used metrics for high-throughput screening data analysis. By favoring false negative over false positive interactions, high-confidence interactomes are produced. Compared to similar, smaller-scale efforts [2,39], our screening is not biased by upfront target prediction that often limits the focus to canonical binding events. The power of this unbiased approach is apparent from the fact that 21% of the identified interactions do not have a seed-match, although it can’t be ruled out that this fraction is enriched for screening false positives.

The validity of our approach is illustrated by numerous observations, such as the high technical and biological reproducibility. Furthermore, interactions identified are strongly enriched for previously established as well as predicted interactions. The successful abrogation of regulation for selected interactions upon binding site mutation further underscores the quality of the interactomes. In general, regulatory miRNAs identified also induce higher down-regulation of endogenous mRNA levels, confirming that reporter gene results can be recapitulated on native transcripts. Moreover, it suggests that regulation at least in part occurs through the induction of mRNA decay, which is in line with published mechanistic models of miRISC effector function [8–10].

In contrast with AGO CLIP-seq and AGO-CLASH data sets, the interactome data set captures the regulatory effect of miRNAs, enabling the study of binding site potency. Interestingly, we found that miRNA interactomes identified here appear to be primarily driven by canonical binding site interactions. While non-canonical offset 6mer, and seed-mismatched or G:U wobble sites also confer regulatory activity, it is clearly less pronounced. Of note, this data set does not hold information on the occurrence and position of miRISC binding events. Hence, no distinction can be made between non-functional and functional binding sites that interact with miRISC. Therefore, the presence of non-functional sites potentially causes us to underestimate the potency of the functional fraction, as we consider them in the same analyses. Functional binding sites have gone through a process of evolutionary selection and potentially require additional unknown sequential or non-sequential features. Each nucleotide pattern, however, also has a baseline prevalence in the genome, without any evolutionary constraint necessarily being involved. The underestimation of potency is therefore expected to be more pronounced for shorter binding site patterns with a higher baseline prevalence, such as non-canonical offset 6mer, seed-mismatched or G:U wobble sites. This objection aside, it has been described that non-canonical sites confer less regulatory activity [19,20,25], and in this respect our data confirms current views. Their more subtle effects might be evolutionary selected to enable expression fine-tuning or they might represent weaker evolutionary intermediates of canonical binding sites. Alternatively, such sites might serve other functions than expression regulation, such as sponge-like miRNA sequestration by the target [40–42]. Correspondingly, the observed hierarchy of canonical binding sites, with increasing potency with pattern length, might in theory also be due to a higher baseline prevalence for 6mer sites, compared to 7mer and 8mer sites, respectively. However, this hierarchy has previously been well-established [15]. A similar reasoning applies to the observed increased regulatory potential in the presence of multiple canonical binding sites, that might reflect a higher chance on the presence of at least one functional site or, alternatively, be a consequence of additive and cooperative interactions between multiple sites, as previously shown to occur [15,43,44].

Nevertheless, even despite potential underestimation of the extent of their effect, our observations confirm that both canonical and non-canonical sites can confer regulatory activity and reduce protein levels. Furthermore, this regulatory activity is clearly enhanced in the presence of additional base pairing with the 3’ end of the miRNA. For canonical and offset 6mer sites this additional effect is pronounced, whereas for G-bulge and seed-mismatched or G:U wobble sites it is moderate. Although demonstrated for canonical [15] and seed-mismatched or G:U wobble sites [13,23], for offset 6mer and G-bulge sites we show this for the first time here. Moreover, G-bulge sites even only seem to have regulatory activity in the presence of 3’ supplementary binding and are inert in its absence. G-bulge sites were initially described for mmu-miR-124 in mice brain, but have not been described as a general mode of miRNA interaction. Recent data indeed suggest that the mode of interaction is highly miRNA-dependent, with different miRNAs preferring different binding site types [28]. It is therefore possible that G-bulge interactions are restricted to a limited subset of miRNAs. Given the limited number of mRNAs considered, functional G-bulge interactions are therefore potentially underrepresented in our data set, precluding robust assessment of their potency. Nonetheless, the regulatory effect upon 3’ supplementary pairing can be demonstrated.

The miRNA interactome data set represents an interesting opportunity for improving miRNA target prediction. Data sets that have typically been used for training prediction models include microarray and SILAC mass spectrometry gene expression measurements after miRNA modulation, as well as AGO HITS-CLIP data. Typically, in these data sets the effects of only one or a couple of miRNAs on a large number of genes are probed. Therefore, resulting models are biased towards a very limited number of miRNAs, making it more difficult to generalize their predictions. Indeed, it has been shown that the mode of interaction can be very miRNA-dependent, with different miRNAs interacting with different binding site types [28]. The miRNA interactome data set, on the other hand, includes interaction information for 470 miRNAs and a limited number of genes. It therefore forms a unique and complementary alternative to currently available data sets. The miRNA interactome data set has already been used as a training data set for building the miSTAR model [37]. This model was trained without considering the non-canonical binding site information in the data set, leaving large potential still unexploited. Nonetheless, the miSTAR model already outperforms published and widely used models, underscoring the quality and the value of the miRNA interactome data set presented here.

## Conclusions

In conclusion, we generated miRNA interactomes for a selection of prominent and widely studied cancer genes by application of a high-throughput reporter screening and introduced a new and simple method for analysis of high-throughput screening data, aimed at eliminating treatment-specific bias.

With this unprecedented and unbiased effort, we realize a four-fold increase in knowledge on regulatory miRNAs for the genes under investigation. This rich and unique resource of interactions will further help unraveling the regulatory networks and dynamic regulation of cancer genes in multiple cancer entities. Notably, the interactome data set provides further insight in the architecture of the effective miRNA interaction and shows the regulatory potential of both canonical and non-canonical binding sites, with the latter being clearly less potent. In addition, it reveals enhanced regulatory activity of both canonical and non-canonical binding sites with 3’ supplementary pairing.

## Materials & methods

### 3’ UTR reporter screening

HEK293T cells were obtained from the American Type Culture Collection (ATCC). Cells were seeded (10,000 cells/well) in opaque 96-well plates in 80 μl RPMI-1640 supplemented with fetal calf serum (FCS) (10%), L-Glutamine (2 mM), and HEPES (25 mM). MicroClime Environmental Lids (Labcyte) filled with 2.5 ml H_2_O were used to minimize edge effects on assay results, due to greater evaporation in edge wells of assay plates. Cells were grown at >90% H_2_O saturation and 5% CO_2_. Twenty-four hours after seeding, cells were co-transfected with 100 ng of a 3’ UTR reporter construct, 20 ng of a control reporter construct and 2.5 pmol of miRNA mimic from a library containing all human mature miRNAs (470) catalogued in release 9.2 of miRBase except for hsa-miR-122a (Ambion’s Pre-miR miRNA Precursor Library—Human V3). Mature miRNA sequences and accession numbers of the mimics are listed in S1 File. Four non-targeting miRNA treated controls (Ambion’s Pre-miR Negative Control #2—AM17111) and four vehicule treated controls were included in each culture assay plate. The 3’ UTR reporter construct is a modified version of the pGL4.11[luc2P] vector (Promega) and contains a multiple cloning site (MCS) upstream of the firefly (Photinus pyralis) luciferase gene (luc2P) that harbors an hPEST protein destabilization sequence. A constitutive RPL10 promotor was cloned in the MCS, and an additional MCS (with XbaI, NheI, AvrII, EcoRV, XhoI and FseI restriction sites) was inserted downstream of the luc2P gene to enable cloning of 3’ UTR sequences. A reporter construct sequence map is provided in S2 File. Human 3’ UTR insert sequences for 17 selected cancer genes are listed in S3 File. As control reporter construct, the pRL-TK vector (Promega) was used, containing a non-regulable sea pansy (Renilla reniformis) luciferase gene (Rluc). Lipid-based co-transfections were performed using 0.4 μl of DharmaFECT Duo transfection reagent (Dharmacon). Transfection mixes with a total volume of 10 μl were incubated for 30 minutes after reconstitution, subsequently diluted two-fold in RPMI-1640, and finally added to cells for a total culture volume of 100 μl. Liquid handling for co-transfection was done using an EVO 100 pipetting robot (Tecan).

Forty-eight hours post-transfection, luc2P and Rluc reporter gene activities were assayed using the Dual-Luciferase Assay System (Promega) according to the manufacturer’s protocol with minor adjustments (LARII and Stop & Glo buffer volumes were reduced to 50 μl). Luminescence values were measured using a FLUOstar OPTIMA microplate reader (BMG LABTECH). A reporter screening spans six 96-well assay plates per gene (and a single gene is assayed per assay plate). Reporter screens were replicated in at least two independent experiments for each gene.

### Interaction score calculation

Cancer gene 3’ UTR reporter (luc2P) activities were normalized to control reporter (Rluc) activities. Normalized reporter activities (NRA) were log_2_-transformed to obtain a symmetrical distribution and expressed as robust z-scores (z), calculated per assay plate, in order to exclude plate-specific bias and compare the results from different assay plates. Robust z-scores were corrected for treatment-specific systematic effects by median centering z-score distributions on a per miRNA basis (S2 Fig). The resulting metric is termed an interaction score (i), and is more negative for miRNAs that interact with the 3’ UTR. Interaction scores from replicated screening s are averaged.

1. $$\text{normalized}\;\text{reporter}\;\text{activity}={\text{NRA}}_{\text{mgr}}={\text{log}}_{\text{2}}{\left(\frac{\text{luc2P}\;\text{activity}}{\text{Rluc}\;\text{activity}}\right)}_{\text{mgr}}$$
2. $$\text{z-score}={\text{z}}_{\text{mgr}}=\frac{{\text{NRA}}_{\text{mgr}}-\text{median(}{\text{NRA)}}_{\text{p}}}{\text{MAD}{\left(\text{NRA}\right)}_{\text{p}}}$$
3. $$\text{interaction}\;\text{score}={\text{i}}_{\text{mgr}}={\text{z}}_{\text{mgr}}-\text{median}{\text{(z)}}_{\text{m}}$$
4. $$\text{average}\;\text{interaction}\;\text{score}=\frac{\sum_{r=1}^{t}i_{mgr}}{t}$$

with m = miRNA; g = gene; r = screening replicate; t = total number of screening replicates for gene g; p = assay plate in which the combination of miRNA m and gene g is probed in screening replicate r (all combinations probed within the same assay plate p involve the same gene g); MAD = median absolute deviation.

In order to establish an interaction score cutoff that discriminates between true positive and true negative interactions with optimal precision, sensitivity and specificity, ROC-curve analysis was performed (Fig 2A). To this purpose, a set of validated interactions was obtained by curating literature, using an automated text-mining approach similar to the one used for the creation of the PubMeth database [45]. Briefly, NCBI’s PubMed database was queried on December 18, 2012 with the names of all miRNAs in the mimic library, their aliases and textual variants (from miRBase and GeneCards), in combination with all aliases and textual variants of the genes under investigation (from GeneCards). PubMed records were subjected to expert revision, with the criterion for inclusion as a true interaction being a successful 3’ UTR reporter assay in which the complete or partial human 3’ UTR sequence was cloned, complemented with a rescue of reporter regulation upon binding site mutation or deletion, or alternatively, omission of the complete 3’ UTR. A total of 92 validated interactions was retrieved for the 17 cancer genes under investigation. An overview of PubMed IDs for publications reporting on these interactions is given in S3 Table. Validated negative interactions are generally not published. As an alternative, a set of interaction scores was generated by duplicate screening of the miRNA library on a reporter gene construct that contained no 3’ UTR.

### Site-directed mutagenesis of reporter constructs

Mutagenesis of 3’ UTR reporter constructs was carried out using the QuikChange II Site Directed Mutagenesis Kit (Stratagene), according to manufacturer instructions (with 30 ng reporter construct input in a 12-cycle PCR reaction). Putative canonical binding sites for 10 selected interactions were mutated, altering nucleotides across positions 3, 4, 5 and 7 of the miRNA’s 5’ end for 6mer and 7mer-A1 sites, and nucleotides across positions 4, 5, 6 and 8 for 7mer-m8 and 8mer sites. More specifically, mutations of A to C, G to T, C to A and U to G were introduced. Mutagenesis primers are listed in S4 Table.

### RT-qPCR screening

HEK293T cells were seeded as described for the 3’ UTR reporter screening. Twenty-four hours after seeding, cells were transfected with 2.5 pmol of miRNA mimics from a miRBase 9.2 library, as described for the reporter screening, but excluding reporter constructs, and using the DharmaFECT2 transfection reagent (Dharmacon). Four non-targeting miRNA treated controls (Ambion’s Pre-miR Negative Control #2—AM17111) and four vehicule treated controls were included in each culture assay plate. Transfections were replicated in two independent experiments.

In order to prepare cDNA from more than 1000 cell culture samples, an approach that we previously validated and in which cDNA synthesis is carried out on crude cell lysates instead of on purified RNA samples was followed [46]. Forty-eight hours after cell seeding, cell cultures were lysed and lysates were DNase and proteinase K treated using the SingleShot Cell Lysis Kit (Bio-Rad), according to the manufacturer’s protocol. Subsequently, cDNA was prepared from 4 μl unpurified cell lysate using the iScript cDNA synthesis kit (Bio-Rad), according to the manufacturer’s protocol.

qPCR gene expression quantifications were performed and reported according MIQE guidelines (Minimum Information for Publication of Quantitative Real-Time PCR Experiments) [47]. Reactions contained 2.5 μl Sso Advanced SYBR mix (Bio-Rad), 1.25 pmol of both forward and reverse primer, and 2 μl of 4x diluted cDNA sample, for a total volume of 5 μl. Thermal cycling conditions were as follows: 95°C for 2 min, followed by 44 cycles of 95°C for 5 sec, 60°C for 30 sec, and 72°C for 1 sec. Melting curve analysis was performed with the following cycling conditions: 95°C for 5 sec, 60°C for 1 min, gradual heating to 95°C at a ramp-rate of 0.11°C/sec, and cooling to 37°C for 3 min. Single replicate reactions were performed in 384-well plates using a CFX384 instrument (Bio-Rad). Liquid handling was done using an EVO 100 pipetting robot (Tecan). All qPCR assays were designed and validated in silico using the primerXL evaluation pipeline [48] and empirically validated, checking both primer efficiency and specificity. Primer sequences are provided in S5 Table, together with information on which transcript isoforms are detected.

Expression levels were normalized, inter-run calibrated, calculated relative to the average expression level in all samples and log_2_-transformed. All calculations were done using the qbase+ software version 2.6 (Biogazelle) [49]. Normalization was performed using four stably expressed reference genes (HPRT1, TBP, UBC and YWHAZ) validated using the geNorm [50] module in qbase+. Inter-run calibration was performed using four calibrator samples included in quadruplicate reactions in each RT-qPCR assay plate. Calibrator samples comprised the MicroArray Quality Control RNA sample A (MAQCA) [51], and a sample consisting of equal mass equivalents of MAQCA RNA, pooled RNA from a neuroblastoma cell line panel (IMR-32, NGP, SK-N-AS, SK-N-SH), and from a T-ALL cell line panel (Jurkat, LOUCY, HPB-ALL, ALL-SIL). Both samples were used in two concentrations with a two-fold difference (2.5 ng and 5 ng cDNA input in qPCR reactions).

### miRNA interaction prediction

Six different models were used to predict miRNA-3’ UTR interactions in the interactome data set: TargetScan (version 6.2) [15,52], miRanda (August 2010 version) [52], MirTarget2 [53], RNA22 (version 1) [54], PITA [55] and DIANA-microT-CDS [32]. Custom predictions (i.e. for the specific miRNA mimic sequences and 3’ UTR reporter vector insert sequences) were performed either online (TargetScan, RNA22), by executing the source code (miRanda, PITA) or offline by the authors from the original paper (MirTarget2, DIANA-microT-CDS). Although most models produce continuous prediction scores, this continuous information was not taken into account. Instead, each miRNA-3’ UTR combination was labeled as either predicted or not predicted to interact by applying the default prediction score cutoff (if any) used by the respective online web tools. In other words, combinations returned by the web tool—or would have been in case of offline prediction—are considered as predicted interactions. An overview of predictions is presented in S6 Table.

### Data mining and statistics

All statistical analyses and data processing steps, including interaction score calculation, were performed using the R statistical programming environment (version 3.0.2).

### miRNA nomenclature and annotation

In this study, we consider miRNA sequences (and miRNA families) annotated in release 9.2 of the miRBase database. Accordingly, we use nomenclature of this release to report and discuss the results. However, when referring to other studies in the discussion, we use the nomenclature applied in these studies. In order to facilitate comparison and integration of the data presented here with other studies, we refer to miRBase Tracker (www.mirbasetracker.org), an in-house developed web tool for miRNA reannotation that enables straightforward assessment of annotation changes between releases [56]. Of note, the most recent miRBase release at time of publication (release 21, June 2014) contains 2588 human mature miRNAs. Compared to miRBase release 9.2, 2124 mature miRNAs are newly annotated, whereas 7 are deleted. A total of 159 miRNAs have an altered canonical sequence and 322 have undergone a name change. An overview of mature miRNA annotation changes between miRBase release 9.2 and 21 is provided in S7 Table.

## Supporting information

S1 Fig miRNA binding sites. **(A)** Canonical 6mer, 7mer-A1, 7mer-m8 and 8mer binding site patterns and the hierarchy in potency. **(B)** Canonical binding sites with 3’ supplementary binding have at least 3 contiguous pairs centered around nucleotides 13 to 16 in addition to a seed-match. Similarly, 3’ compensatory binding involves at least 4 contiguous pairs centered around nucleotides 12 to 17 and compensates for incomplete seed-matches or G:U wobbles. **(C)** Offset 6mer sites match nucleotides 3 to 8 of the 5’ end of the miRNA. **(D)** Seed-mismatched or G:U wobble sites have a mismatch that can occur at any position within the seed region. **(E)** G-bulge sites bulge out a guanosine between the nucleotides across positions 5 and 6 of the miRNA in order to match the miRNA seed region. Adapted and reprinted from Van Peer et al. [37] under a CC-BY 4.0 license, with permission from Oxford University Press, original copyright 2016.
(TIFF)

S2 Fig Z-scores versus interaction scores. **(A)** Boxplot distributions of z-scores for each miRNA, with ordering along the x-axis according to increasing median z-score. **(B)** Boxplot distributions of interaction scores for each miRNA.
(TIFF)

S3 Fig Cancer gene-miRNA interactomes. The miRNA interactomes of **(A)** ALK, **(B)** BRCA1, **(C)** BRCA2, **(D)** EZH2, **(E)** FBXW7, **(F)** HRAS, **(G)** MYB, **(H)** MYC, **(I)** MYT1L, **(J)** NOTCH1, **(K)** PALB2, **(L)** PHF6, **(M)** PHOX2B, **(N)** RB1 and **(O)** ZEB2.
(TIFF)

S1 Table PMIDs cancer gene selection. PubMed IDs (PMIDs) of publications describing the involvement of the cancer genes under study in different cancer entities.
(XLSX)

S2 Table 3’ UTR reporter and RT-qPCR screening results. Results for all 7990 miRNA-3’ UTR combinations probed in replicate 3’ UTR reporter and RT-qPCR screenings. For each cancer gene under study, the identified miRNA interactome is listed.
(XLSX)

S3 Table PMIDs established interactions. PubMed IDs (PMIDs) of publications describing established miRNA interactions for the cancer genes under study.
(XLSX)

S4 Table Site-directed mutagenesis primers. Primer sequences for site-directed mutagenesis of canonical binding sites in 3’ UTR reporter constructs for the cancer genes under study.
(XLSX)

S5 Table RT-qPCR primers. Forward and reverse primer sequences for reference genes and the cancer genes under study in the RT-qPCR screening.
(XLSX)

S6 Table Predicted miRNA-3’ UTR interactions. Overview of predicted miRNA-3’ UTR interactions in the interactome data set.
(XLSX)

S7 Table miRBase release comparison. Overview of mature miRNA annotation changes between miRBase release 9.2 and 21.
(XLSX)

S1 File miRNA sequences. FASTA file containing miRNA sequences annotated in miRBase 9.2 (except for hsa-miR-122a). Sequence identifiers contain the mature miRNA accession number and name.
(TXT)

S2 File 3’ UTR reporter vector map. Modified pGL4.11[luc2P] 3’ UTR reporter vector sequence map, with indication of all functional elements.
(TXT)

S3 File 3’ UTR sequences. FASTA file with 3’ UTR sequences for the cancer genes under study, cloned in the modified pGL4.11[luc2P] reporter vector.
(TXT)
