# Supplementary figures and images for: A high-throughput 3’ UTR reporter screening identifies microRNA interactomes of cancer genes
Source: PLoS One. 2018 Mar 9;13(3):e0194017. doi: 10.1371/journal.pone.0194017 (PMC5844555; doi:10.1371/journal.pone.0194017)

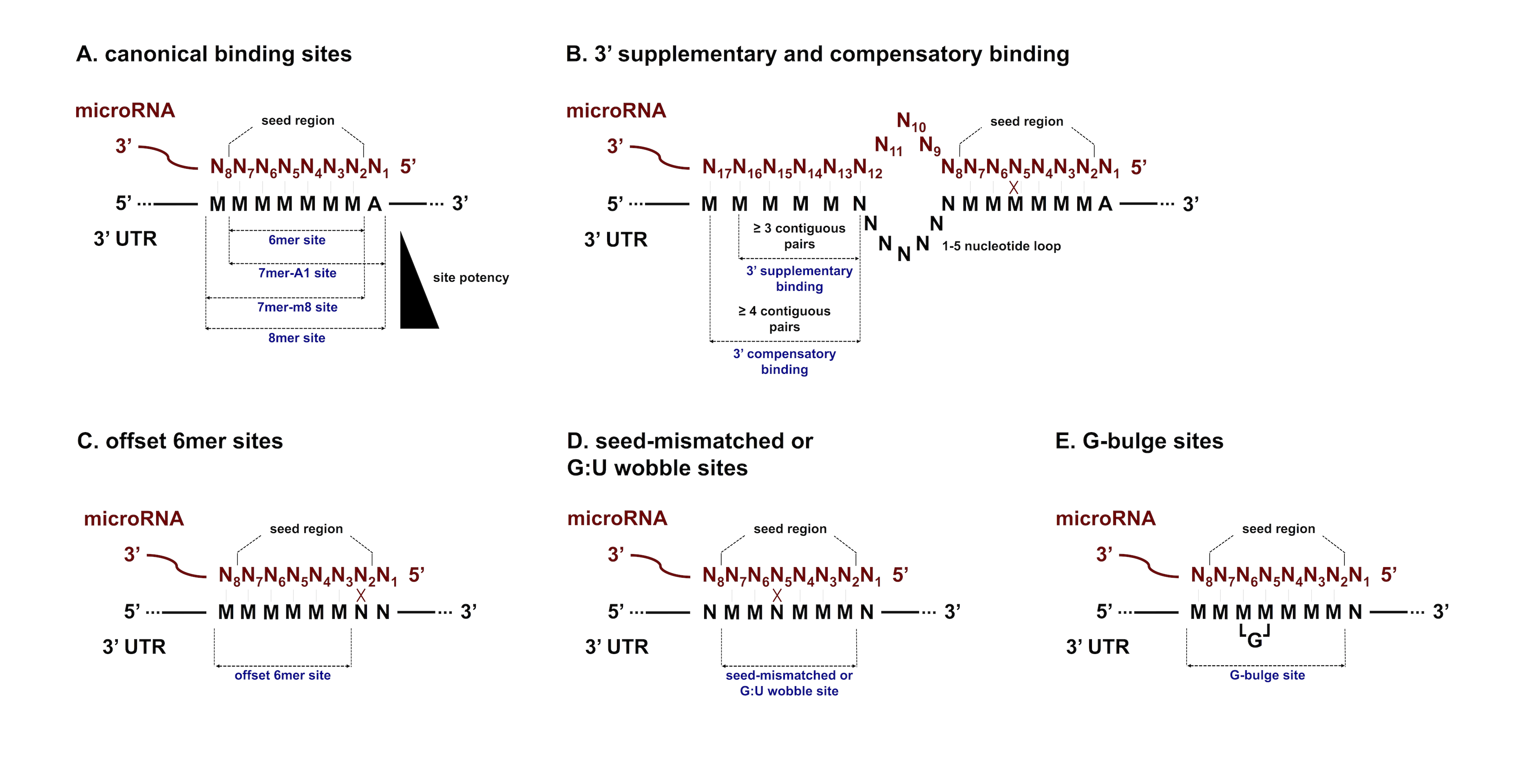

Supplement: S1 Fig — (A) Canonical 6mer, 7mer-A1, 7mer-m8 and 8mer binding site patterns and the hierarchy in potency. (B) Canonical binding sites with 3’ supplementary binding have at least 3 contiguous pairs centered around nucleotides 13 to 16 in addition to a seed-match. Similarly, 3’ compensatory binding involves at least 4 contiguous pairs centered around nucleotides 12 to 17 and compensates for incomplete seed-matches or G:U wobbles. (C) Offset 6mer sites match nucleotides 3 to 8 of the 5’ end of the miRNA. (D) Seed-mismatched or G:U wobble sites have a mismatch that can occur at any position within the seed region. (E) G-bulge sites bulge out a guanosine between the nucleotides across positions 5 and 6 of the miRNA in order to match the miRNA seed region. Adapted and reprinted from Van Peer et al. [37] under a CC-BY 4.0 license, with permission from Oxford University Press, original copyright 2016. (TIFF) [file pone.0194017.s001.tiff]

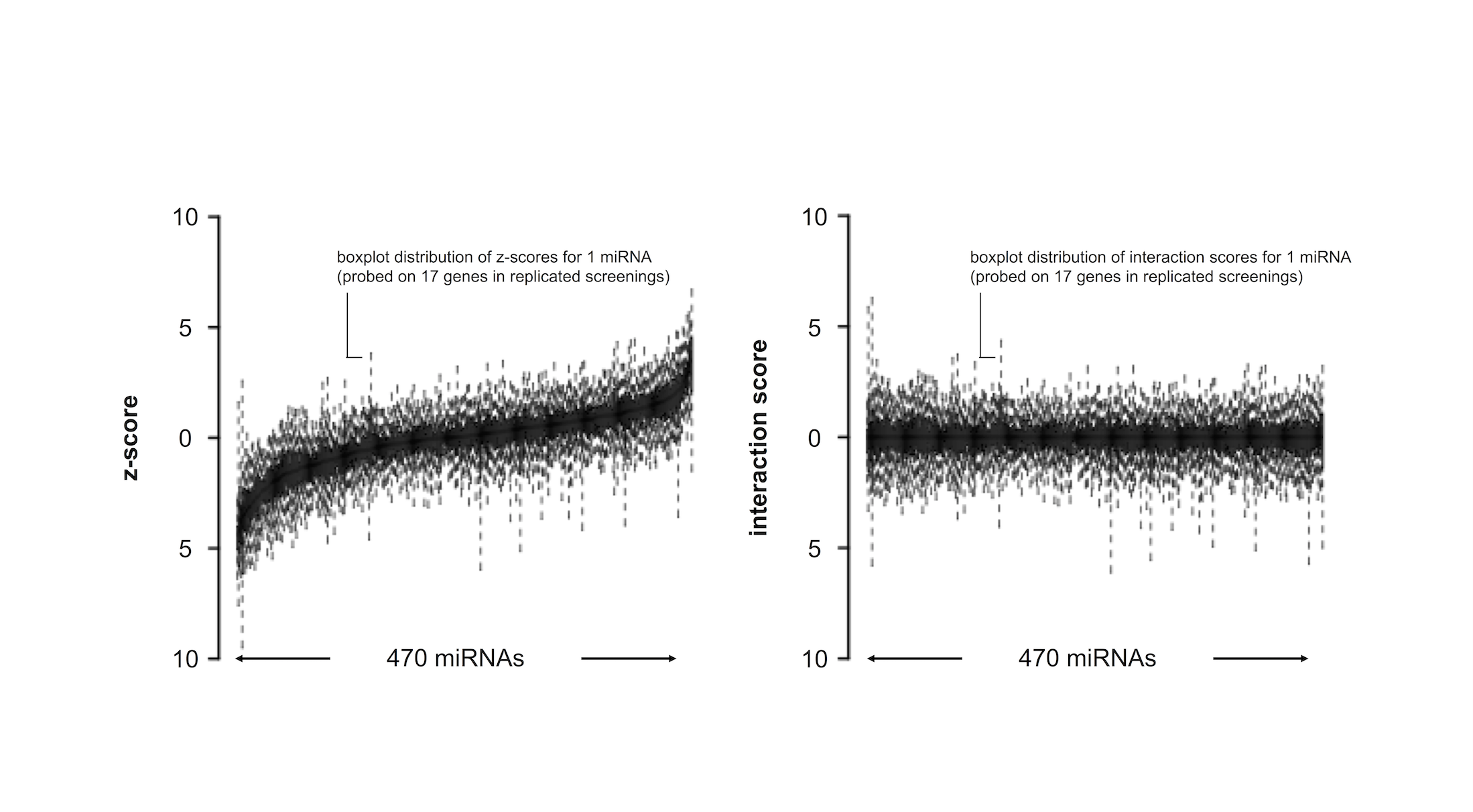

Supplement: S2 Fig — (A) Boxplot distributions of z-scores for each miRNA, with ordering along the x-axis according to increasing median z-score. (B) Boxplot distributions of interaction scores for each miRNA. (TIFF) [file pone.0194017.s002.tiff]
